# P4HA1 is a new regulator of the HIF-1 pathway in breast cancer

**DOI:** 10.15698/cst2019.01.173

**Published:** 2019-01-09

**Authors:** Ren Xu

**Affiliations:** 1 Markey Cancer Center, University of Kentucky, Lexington, KY 40536, USA.; 2 Department of Pharmacology and Nutritional Sciences, University of Kentucky, Lexington, KY 40536, USA.

**Keywords:** collagen, hydroxylation, α-ketoglutarate, cancer stem cell, chemoresistance

## Abstract

Hypoxia-Inducible Factor (HIF)-1 is a transcription factor that plays the key role in response to low oxygen concentrations, or hypoxia. Activation of the HIF-1 pathway is not only crucial for normal tissue development and function, but also involved in disease progression, such as cancer. Cancer cells proliferate rapidly in solid tumors, and thus, solid tumors consume much more oxygen and nutrients than normal tissue and generate oxygen tension. It is well established that oxygen tension in solid tumor tissue induces the aberrant activation of the HIF-1 pathway, and subsequently promotes angiogenesis and tumor progression. Breast cancer is a heterogeneous disease and can be classified into luminal, Her2 positive, and triple negative (TNBC) subtypes base on ER/PR and Her2 expression in the breast cancer tissue. HIF-1 activation is observed in all breast cancer subtypes; interestingly, the HIF-1 pathway is hyperactivated in TNBCs compared to other subtypes. The differential activation of the HIF-1 pathway in breast cancer subtypes suggests that oxygen-independent pathways may be involved in HIF-1 regulation during TNBC progression. However, these pathways have not been well-characterized. We demonstrate that collagen proly 4 hydroxylase 1 (P4H1) induces HIF-1α expression at the protein level by modulating α-ketoglutarate (α-KG) and succinate levels. These results reveal a novel link between collagen hydroxylation and activation of the HIF-1 pathway and unveil a new HIF-1 regulation mechanism in TNBC.

Prolyl hydroxylation is a very common posttranslational protein modification. About 4% amino acid residues in animal proteins are hydroxyproline, and most of hydroxyproline is within collagen. Regulation of protein stability and folding is the two major functions for prolyl hydroxylation. For instance, the hydroxylation on proline 402 (Pro402) and 564 (Pro564) induces ubiquitination and degradation of HIF-1α, and the hydroxylation is regulated by a family of oxygen-dependent dioxygenases (PHD). Proper folding and formation of the collagen triple helix requires 4-hydroxylation of proline on the GPX repeat region, which is catalyzed by Collagen P4H (cP4H).

CP4H is a α2β2 tetrameric α-KG-dependent dioxygenase, and succinate is a product of the hydroxylation reaction. Three α subunits (P4HA) of cP4H have been identified in mammalian cells, and they are responsible for both peptide binding and for catalytic activity. P4HA1 is the major isoform and contributes the majority of prolyl 4-hydroxylase activity in most cell types and tissues. Recently, studies showed that expression of P4HA1, 2 and 3 is induced during cancer development and progression, and that increased P4HA expression correlates with poor prognosis in cancer patient. Importantly, P4HA1 expression in cancer cells is required for breast cancer metastasis. However, we know little about how expression of P4HA1 in cancer cells promotes tumor progression.

Using unbiased gene coexpression analysis, we have identified the HIF-1 pathway as a potential downstream target of P4HA1. CP4H has three-fold higher affinity to α-KG and six-fold higher affinity to oxygen compared to PHD, and about 38% of proline in collagen are hydroxylated. Therefore, increased expression of cP4H may compete with PHD to consume a substantial amount of α-KG (**Figure 1**). Indeed, we found that cytoplasmic α-KG levels are reduced in P4HA1-expressing cells. Rescue experiments further confirm the hypothesis that P4HA1 regulates HIF-1α stability by modulating α-KG and succinate levels. Regulation of HIF-1α stability with small metabolites has also been demonstrated in glioma. Mutation of IDH-1 in glioma induces the HIF-1 pathway by reducing α-KG production. Our results suggest that metabolites such as α-KG and succinate are regulated by cP4H1 in TNBC, which in turn contributes to HIF-1 activation during breast cancer development and progression. It has been shown that lysyl oxidase (LOX) expression also increases HIF-1α protein levels. Similar mechanisms may mediate the crosstalk between LOX and HIF-1α. In addition, P4HA1 expression may modulate activity of other α-KG-dependent enzymes, such as TET enzymes and JMJD histone demethylases.

**Figure 1:**
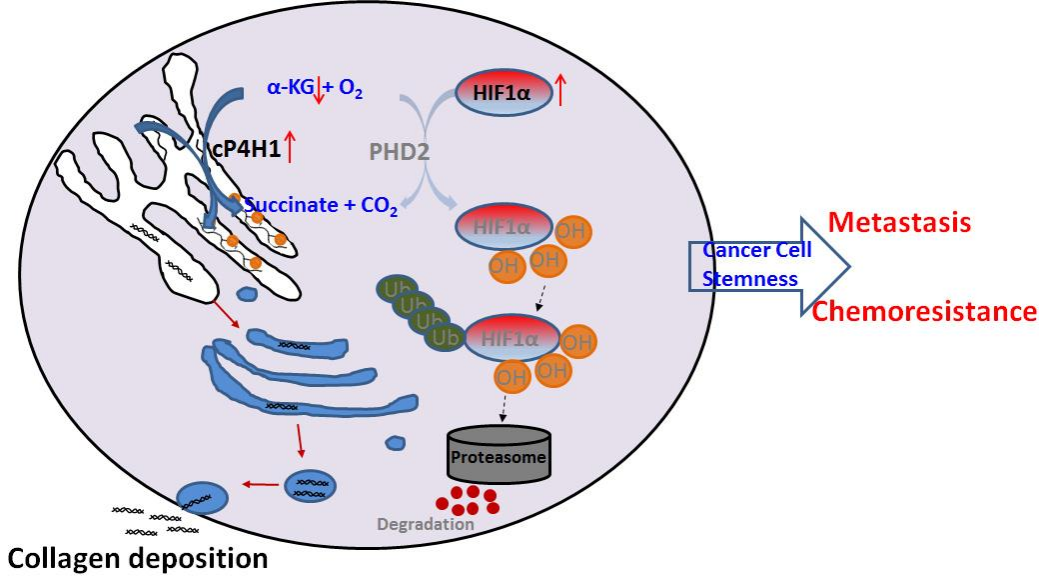
FIGURE 1: A scheme showing the molecular mechanism by which cP4H1 enhances HIF-1stability and cancer cell stemness in TNBC.

HIF-1 pathway is hyperactivated in TNBC and is associated with poor prognosis. We found that expression of the HIF-1 gene signature correlates with P4AH1 levels in breast cancer tissue samples. Tissue microarray data showed that P4HA1 protein expression is significantly upregulated in TNBC compared to ER positive breast cancer. These results indicate that activation of the HIF-1 pathway in TNBC is at least partially regulated by the P4HA1. However, other mechanisms may also contribute to the HIF-1 activation in TNBC. For instance, XBP1 interacts with HIF-1α and enhances its transcription factor activity and subsequently drives TNBC tumorigenicity. In addition, SHARP1 suppresses breast cancer metastasis by promoting proteasomal degradation of HIFs.

Activation of the HIF-1 pathway is crucial for the survival and self-renewal of stem cells. Cancer stem cells are the driver of cancer progression and chemoresistance. Interestingly, P4HA1 expression is upregulated in cancer stem cell enriched cell population. We further demonstrated that P4HA1 regulates cancer cell stemness through the HIF-1 pathway. Importantly, silence or inhibition of P4HA1 activity sensitizes TNBC to chemotherapeutic agents *in vitro* and *in vivo*. Moreover, high levels of P4HA1 are associated with chemoresistance in TNBC patients. These results indicate that targeting P4HA1 is a promising strategy to inhibit cancer stem cells and overcome chemoresistance in TNBC.

In summary, our findings fill a critical gap in our knowledge about the regulation of the HIF-1 pathway and tumor-initiating cells during breast cancer progression. Given that activation of HIF-1 and expression of P4HA1 are particularly robust in TNBC, inhibition of P4HA1 is a promising strategy for blocking TNBC progression. Therefore, it is crucial to identify specific P4HA1 inhibitor in the future.

